# Identification of crucial genes and possible molecular pathways associated with active vitamin D intervention in diabetic kidney disease

**DOI:** 10.1016/j.heliyon.2024.e38334

**Published:** 2024-09-25

**Authors:** MingXia Zhang, Mi Tao, Quan Cao, Yousheng Cai, Lin Ding, Zhenni Li, Wen Chen, Ping Gao, Lunzhi Liu

**Affiliations:** a Department of Nephrology, Minda Hospital Affiliated to Hubei Minzu University, Hubei Clinical Research Center for Kidney Disease, Enshi, China; b Department of Nephrology, Zhongnan Hospital, Wuhan University, Wuhan, China; c Department of Nephrology, Zhongnan Hospital of Wuhan University, School of Pharmaceutical Sciences, Wuhan University, Wuhan, 430071, China

**Keywords:** Calcitriol, Diabetic kidney disease, Fibrosis, Biomarkers, FoxO pathway

## Abstract

**Background:**

A significant cause of advanced renal failure is diabetic nephropathy (DKD), with few treatment options available. Calcitriol shows potential in addressing fibrosis related to DKD, though its molecular mechanisms remain poorly understood. This research seeks to pinpoint the crucial genes and pathways influenced by calcitriol within the scope of DKD-related fibrosis.

**Methods:**

Single-cell gene expression profiling of calcitriol treated DKD rat kidney tissue and screening of fibrosis-associated cell subsets. Mendelian randomization and enrichment analyses (CIBERSORT, GSVA, GSEA, Motif Enrichment) were used to explore gene-immune cell interactions and signaling pathways. Key findings were validated using independent datasets and protein expression data from the Human Protein Atlas.

**Results:**

Calcitriol treatment reduced proliferative cell populations and highlighted the FoxO signaling pathway's role in DKD. *SUMO3* and *CD74* were identified as key markers linked to immune infiltration and renal function. These genes were significantly associated with creatinine levels and eGFR, indicating their potential role in DKD progression.

**Conclusion:**

Our results suggest that calcitriol modulates DKD fibrosis through the FoxO pathway, with *SUMO3* and *CD74* serving as potential biomarkers for kidney protection. These results provide fresh insights into strategies for treating DKD.

## 1 Introduction

There is a great concern in the global health community regarding diabetic kidney disease (DKD), a common complication arising from diabetes that significantly contributes to the progression of end-stage renal disease (ESRD) [1]. The condition typically involves several harmful processes, such as an increase in fat levels, long-lasting inflammation, and oxidative stress. Additionally, the buildup of certain proteins outside cells contributes to the gradual worsening of kidney function, eventually leading to ESRD. DKD also significantly elevates the risk of cardiovascular and cerebrovascular diseases [2,3]. The complexity and variability of DKD pose major challenges for current treatments. In the absence of effective drug therapies, many patients advance to ESRD, necessitating renal replacement options like dialysis or kidney transplantation [4,5]. Although some medications have shown efficacy in improving DKD, the overall clinical landscape remains largely unchanged [6]. Consequently, there is a pressing need for novel treatment approaches to better control and manage DKD, given its increasing prevalence and profound impact on clinical outcomes.

Active vitamin D has garnered increasing attention due to its multifaceted biological activities, particularly in the context of DKD. The regulation of calcium and phosphate levels, immune response modulation, and anti-inflammatory effects are all critically influenced by active vitamin D [7,8]. Evidence indicates that vitamin D could offer protective benefits in slowing the progression of chronic kidney disease [9]. Early research on DKD suggests that active vitamin D may provide therapeutic advantages by mitigating renal inflammation and preserving kidney function [10,11]. However, despite these promising findings, the specific molecular mechanisms by which active vitamin D impacts DKD fibrosis remain unclear and warrant further investigation.

This study aims to systematically explore the mechanisms by which active vitamin D affects the treatment of diabetic kidney disease (DKD) through an extensive analysis of diverse datasets. Utilizing Mendelian randomization, the research seeks to assess the potential causal relationship between active vitamin D levels and the initiation and progression of fibrosis in DKD. This method reduces the influence of external factors and reverse causality, allowing for a clearer assessment of the causal relationship [12]. Single-cell sequencing technology will be employed to analyze the mechanism of action of active vitamin D in DKD fibrosis, discover new therapeutic targets, and provide a basis for cell-specific intervention strategies, thereby improving therapeutic efficacy [13]. Additionally, bulk RNA sequencing will explore the immune microenvironment and regulatory networks within DKD. This comprehensive study seeks to elucidate the molecular mechanisms of active vitamin D in DKD, providing new scientific evidence for future precision medicine and personalized treatments to enhance patient outcomes and quality of life.

## 2 Methods

### 2.1 GEO data source and preparation

A total of two major datasets, GSE30528 and GSE30529, were retrieved from Gene Expression Omnibus (GEO) repository. Dataset GSE30528, employed as the primary analysis set, includes genetic expression data from kidney tissues of 10 DKD patients and 8 healthy controls. Dataset GSE30529, utilized as an independent validation set, comprises gene expression data from kidney tissues of 12 individuals diagnosed with DKD and 13 non-DKD controls. Supplementary Table 1 provides detailed information about these datasets. Fibrosis-related gene collections were sourced through the GeneCards database (https://www.genecards.org/). Since the dataset are sourced from a public database, approval from a local ethics committee was not required.

### 2.2 Animal and experimental design

Fifteen male Sprague-Dawley (SD) rats, each six weeks old, were sourced from Three Gorges University (Yichang, China). The rats were housed in a pathogen-free (SPF) environment with tightly regulated conditions, stabilizing the temperature at 22 °C and relative humidity at 50 % and a 12-h cycle of light and darkness. The animals were provided with unlimited availability of food and water for the entire study period, ensuring their nutritional needs were met without restriction. The Animal Experiment Ethics Committee of Minda Hospital, affiliated with Hubei Minzu University, reviewed and approved the study protocol (Ethics Approval Number: Y2022009).

The rats were randomly distributed into one of three experimental groups, each consisting of five animals. The first group, designated as the wild-type (WT) control, was administered an intraperitoneal injection of sodium citrate buffer (25 mM/L, pH 4.0) and maintained on a regular chow diet. The other rats were fed a diet rich in fats and glucose for eight weeks to induce metabolic alterations. Following this period, the rats were administered an intraperitoneal dose of 30 mg/kg streptozotocin (STZ) to develop a model of DKD. The DKD rats were further divided into two subgroups (n = 5 per subgroup): the DKD model group and the DKD + calcitriol group. Rats in the DKD + calcitriol group received both STZ injections (60 mg/kg, intraperitoneally) and were treated with calcitriol (0.03 μg/kg/day, orally). Calcitriol was supplied by Hy cell Biotechnology (Wuhan, China).

Glucose concentrations in the blood were measured via tail vein sampling 72 h following the STZ injection. DKD was deemed successfully induced when random blood glucose levels reached or exceeded 16.7 mmol/L. At the end of the 8-week intervention period, each rat was anesthetized with an intraperitoneal administration of sodium pentobarbital (30 mg/kg), followed by euthanasia through cervical dislocation. Subsequently, kidney tissues were harvested and stored at a temperature of −80 °C for subsequent examination and evaluation. For additional molecular analysis, right kidney tissues were obtained from two rats in the diabetic kidney disease cohort and two from the diabetic kidney disease + calcitriol cohort. The samples were pooled according to group and sent to Ruixing Biotechnology (Wuhan, China) for single-cell gene expression profiling.

### 2.3 Single-cell RNA profiling analysis

Kidney tissues were isolated from control rats and experimental groups to generate single-cell suspensions. Two samples from the untreated DKD cohort and two from the calcitriol-administered group were combined into two pooled samples. The kidney cells were subsequently reconstituted in phosphate-buffered saline (PBS, Thermo Fisher Scientific, Waltham, MA, USA) at cell concentrations ranging between 1.6 million and 7 million cells per milliliter, preparing them for sequencing. The cells were then loaded onto 10x Chromium microfluidic chips (10x Genomics, Pleasanton, CA, USA) and barcoded using the 10x Genomics Chromium System. Total RNA was isolated and converted into cDNA, after which a single-cell sequencing library was constructed using the 3′ v2 kit from the Chromium Single Cell system (10x Genomics). Sequencing was conducted on the NovaSeq 6000 system (Illumina, San Diego, CA, USA) utilizing 150 bp paired-end reads.

Filtering and quality assessment for the scRNA-Seq data included eliminating low-quality cells and genes with minimal expression. Data normalization, homogenization, and PCA analysis were performed sequentially. The ElbowPlot method was employed to determine the optimal count of principal components (PCs), and clustering was executed through UMAP. Cell group classifications were assigned using the celldex toolkit, emphasizing the identification of cells relevant to the progression of the disease. Using the LIMMA software for differential gene expression analysis [14], we utilized a corrected p-value cutoff of less than 0.05 to detect meaningful differences.

## 3 Mendelian randomization analysis

### 3.1 Exposure data (pQTL)

Protein quantitative trait loci (pQTL) data were sourced from the 2021 version of the deCODE database (https://www.decode.com/summarydata/). This dataset contains a genome-wide association study (GWAS) of 35,559 European individuals, using 4907 aptamers to quantify plasma protein concentrations. Single nucleotide polymorphisms (SNPs) linked to each gene with a significance threshold of P < 1e-5 were regarded as potential instrumental variables (IVs). To maintain accuracy, linkage disequilibrium (LD) between SNPs was assessed, and strict criteria (R2 < 0.001 and p2 < 5e-5) were implemented to filter for the most relevant SNPs.

### 3.2 Outcome data

Outcome data were obtained from the FinnGen repository (https://www.finngen.fi/en/access_results), a comprehensive genetic research project focused on the European population. We specifically downloaded GWAS data for DKD, comprising 4111 cases and 308,539 controls.

### 3.3 Statistical methods

To establish causal relationships between pQTL and DKD, we used outcome IDs extracted from the FinnGen biobank database. The analysis utilized various statistical techniques to assess causality and determine the robustness of the instrumental variables: MR Egger, Weighted Median, Weighted Mode, and Inverse-Variance Weighted (IVW) methods. The reliability of the causal inferences was reinforced through further statistical validation, including the Wald ratio, offering a detailed assessment of gene expression's influence on DKD.

### 3.4 Sensitivity analysis

A leave-one-out sensitivity analysis was conducted to assess the consistency of the genetic variants' effect on DKD risk. This approach sequentially excludes each SNP and recomputes the combined effect size for the remaining SNPs. By generating a new point estimate and its 95 % confidence interval with each SNP removal, we could evaluate each SNP's individual contribution. This analysis ensures robustness and identifies any disproportionately influential SNPs. Results were summarized in a chart comparing the estimates after individual SNP removals and the overall estimate with all SNPs included.

### 3.5 Heterogeneity test

To evaluate statistical heterogeneity among the studied SNPs, a Mendelian heterogeneity test was performed. This analysis calculated the weighted sum of squares for effect sizes and standard errors of each SNP to produce a Q value. The Q value follows a chi-square distribution, where the degrees of freedom correspond to the total number of SNPs minus one. A p-value exceeding 0.05 suggests no significant heterogeneity in SNP effect sizes, indicating consistent impacts on DKD risk.

### 3.6 Functional exploration of GO and KEGG pathways

The identified genes underwent Gene Ontology (GO) and KEGG pathway enrichment analysis via the clusterProfiler package in R. GO terms were categorized into biological processes, cellular components, and molecular functions, with statistical significance evaluated using an adjusted p-value threshold of <0.05. The results were depicted through bar plots, with color gradients representing the statistical significance of each term or pathway. Data visualization was achieved using ggplot2 to ensure clarity and precision.

### 3.7 Analysis of immune cell infiltration

The composition of immune cells was evaluated using the CIBERSORT algorithm, applying the LM22 signature matrix to the GSE30528 dataset [15]. The GSE30528 dataset was normalized by calculating transcripts per million (TPM), and batch effects were corrected using the ComBat method to improve consistency. To ensure robust estimations of immune cell proportions, CIBERSORT was run with 1000 permutations. The proportions of immune cells between the groups were compared using the Wilcoxon rank-sum test, a method well-suited for handling data that do not follow a normal distribution. All visualizations were generated using the ggplot2 and pheatmap packages in the R programming environment.

### 3.8 Pathway enrichment evaluation using GSEA

To gain insights into the signaling pathway differences among groups with varying gene expression levels, we implemented Gene Set Enrichment Analysis (GSEA). This method utilized the curated gene sets from the MsigDB (version 7.0) database, providing a framework for identifying pathways unique to specific subtypes. By examining the differential expression patterns across these subtypes, we pinpointed key gene sets that were significantly enriched, ranking them by enrichment scores with a corrected p-value cutoff of less than 0.05. GSEA proves particularly useful in linking gene expression profiles to biological processes, making it invaluable for studies that aim to uncover the functional relevance of disease subtypes and their molecular mechanisms.

### 3.9 Gene Set Variation Analysis (GSVA)

Unlike conventional gene set enrichment analysis, GSVA calculates enrichment scores at the individual sample level, enabling the identification of subtle pathway activity changes. To explore the role of key genes in DKD renal tubules, a training dataset was constructed from transcriptomic data, and the GSVA approach was employed. At the outset, samples were categorized into high-expression (HExp) and low-expression (LExp) groups according to the expression levels of the key genes. GSVA was then applied to compute the enrichment scores for specific gene sets within each sample, and differences between the expression groups were assessed, generating t-values to indicate pathway activity changes across various signaling pathways. This approach allows for the detection of biological pathway alterations across samples, providing deeper insights into functional dynamics and uncovering intricate mechanisms underlying the data.

### 3.10 Transcriptional regulation analysis of key genes

Analysis of enriched transcription factor (TF) binding motifs was carried out using the HOMER software (v4.10) with its default settings [16]. The gene set analyzed included key renal tubular genes involved in DKD, derived from RNA-seq data of the DKD training set, which compared renal tubular samples from DKD patients with healthy controls. A background gene set, adjusted for sequence length and GC content, was applied to control for non-specific motif matches. Motif enrichment was evaluated using the cisBP database, with results quantified by normalized enrichment scores (NES) and area under the curve (AUC) values. High-confidence transcription factor annotations were sourced from the cisBP database and supplemented with experimental data when available. Only motifs with significant NES and AUC scores were selected for further analysis.

### 3.11 Clinical Correlation with key genes

Gene expression data for key renal tubular genes were acquired and analyzed from the DKD-specific dataset in NephroseqV5, with the GSE30529 dataset serving as an independent validation cohort. The gene expression data were processed through TPM normalization and log2 transformation, with low-expression genes (TPM <1) being excluded from further analysis. To evaluate the diagnostic relevance of key genes in DKD, ROC curve analysis was performed with the pROC package in R, and the area under the curve (AUC) was computed to gauge their ability to distinguish DKD patients from healthy controls. Correlation analysis was conducted to explore the relationship between gene expression and clinical parameters, such as serum creatinine and estimated glomerular filtration rate (eGFR), using either Pearson or Spearman correlation coefficients depending on the results of the Shapiro-Wilk test for normality. The eGFR values were calculated using both the MDRD and CKD-EPI equations. All statistical analyses and visualizations were performed in R, with data visualized through the ggplot2 package.

### 3.12 Verification of key gene expression by immunohistochemistry

The expression of key genes was validated through immunohistochemistry (IHC) analysis. Data for this validation were sourced from the Human Protein Atlas (HPA) repository (https://www.proteinatlas.org/), a robust resource offering extensive proteomic information. This database contains data generated from 27,520 antibodies, each targeting one of 17,288 human proteins, providing a valuable platform for assessing protein expression patterns in various tissues. This database provides detailed protein atlases at various levels, including subcellular, single-cell, cell line, and tissue, and contains extensive resources of IHC staining images. For this study, we downloaded IHC images of normal kidney tissue for the selected key genes from the HPA database. The expression patterns observed in these images were analyzed to corroborate the findings from our gene expression analysis.

### 3.13 Statistical analysis

Mendelian Randomization (MR) analysis was grounded in three fundamental assumptions. First, the relevance assumption was confirmed by selecting instrumental variables (IVs) with an F-statistic greater than 10, ensuring strong instruments. Second, the independence assumption was evaluated using MR-Egger regression and weighted median analysis to detect and account for potential pleiotropy. Third, the exclusion restriction assumption was tested through sensitivity analyses, ensuring that the IVs influenced the outcome only through the exposure. IVs were drawn from genome-wide association studies (GWAS) based on their significant association with the exposure trait (p < 5 × 10^-8). The primary analysis utilized the inverse-variance weighted (IVW) method, with MR-Egger and weighted median approaches employed for additional robustness. Statistical significance was set at a p-value threshold of 0.05, and adjustments for multiple comparisons were made using the Benjamini-Hochberg false discovery rate (FDR). All analyses were conducted in R version 4.2.2.

## 4 Results

### 4.1 Analysis of scRNA-Seq data at the single-cell resolution

**Fig. 1:**
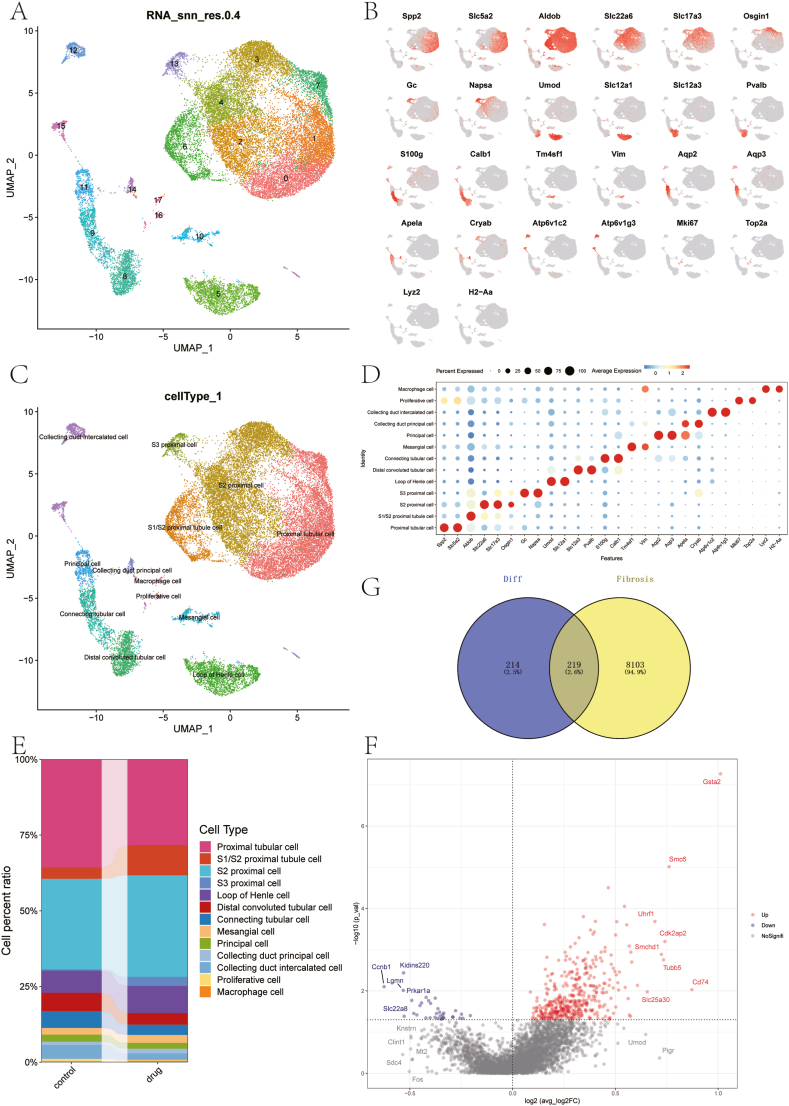
Cell Annotation and Differential Analysis (A) UMAP-based clustering of cells into 18 clusters using PCA components. (B) Scatter plot showing marker gene expression. (C) Annotation of cells into 13 distinct types. (D) Bubble chart illustrating cell types and their associated markers. (E) Differences in cell type proportions between sample groups. (F) Volcano plot depicting differential expression in proliferative cells. (G) Venn diagram showing intersecting genes. UMAP: Uniform Manifold Approximation and Projection; PCA: Principal Component Analysis.

To investigate the cellular mechanisms contributing to DKD and evaluate the effects of Calcitriol treatment, this study employed single-cell transcriptomics as a technique to analyze gene expression at the individual cell level. The experimental setup included two DKD rats treated with Calcitriol and two untreated DKD rats, with renal tissues subjected to scRNA-Seq analysis. The Seurat framework was applied to process the gene expression data, applying filters to remove low-quality cells based on the following criteria: nFeature_RNA greater than 200, nFeature_RNA less than 4000, nCount_RNA below 25000, and percent.mt under 75. This filtering retained 25,841 cells. Post-filtering, gene expression distributions were illustrated using violin and scatter plots (Supplementary Figs. 1A and 1B). Emphasis was placed on the ten genes showing the highest normalized variance (Supplementary Fig. 1C), potentially serving as biomarkers and key regulatory genes. After normalization, principal component analysis (PCA), and harmony analysis (Supplementary Figs. 1D–1F), UMAP analysis was conducted to reveal the spatial relationships among cell clusters, identifying 18 distinct cellular subtypes (Fig. 1A). These results provide crucial insights into the cellular heterogeneity present in DKD kidneys.

### 4.2 Cellular subtype annotation in scRNA-Seq

In this research, we identified and annotated 18 distinct cellular subtypes (Fig. 1B) from scRNA-Seq, organizing them into 13 unique renal cell categories: Proliferative cell, mesangial cell, S3 proximal cell, macrophage cell, S1/S2 proximal tubule cell, Loop of Henle cell, collecting duct intercalated cell, principal cell, S2 proximal cell, proximal tubular cell, distal convoluted tubular cell, collecting duct principal cell, and connecting tubular cell (Fig. 1C). The cellular markers for each type were depicted in a bubble chart (Fig. 1D), and the distribution of each cell type was displayed in a bar chart (Fig. 1E).

Given the established association between proliferative cells and fibrosis [17], the proliferative cell subtype was analyzed from both control and DKD (diabetic kidney disease) + Calcitriol intervention groups to determine differentially expressed genes using a P-value cutoff of less than 0.05. This analysis identified 433 marker genes, which were visualized using a volcano plot (Fig. 1F). Subsequently, fibrosis-related genes sourced from the GeneCards repository were compared with the identified marker genes showing differential expression, yielding 219 common genes. This gene set was used for Mendelian randomization analysis to investigate their potential roles in the fibrotic process (Fig. 1G).

### 4.3 Biological function enrichment analysis

**Fig. 2:**
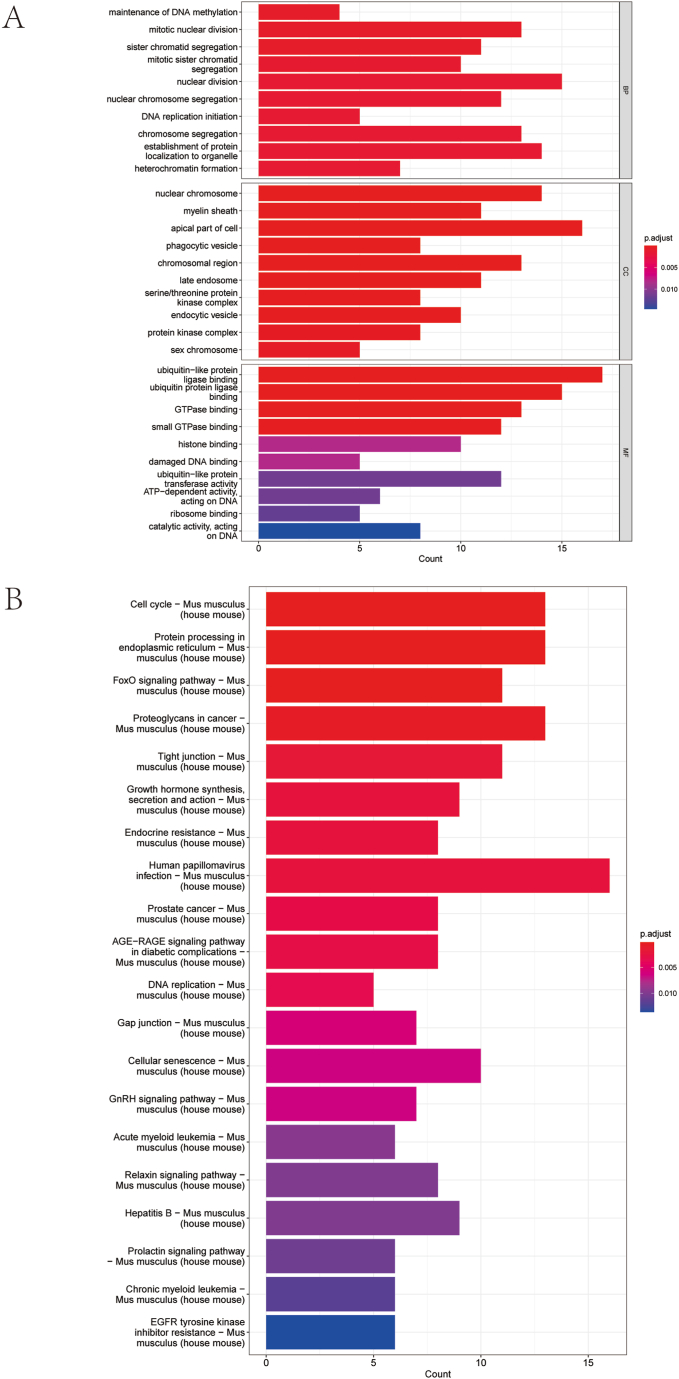
Enrichment Analysis (A) GO enrichment analysis results of intersecting genes. (B) KEGG pathway enrichment analysis results of intersecting genes. GO: Gene Ontology; KEGG: Kyoto Encyclopedia of Genes and Genomes.

To investigate the biological roles of the overlapping genes identified in this research, we performed pathway enrichment analyses utilizing both Gene Ontology (GO) and KEGG databases. The GO analysis revealed a notable enrichment of these genes in pathways linked to mitotic nuclear division, nuclear division, and nuclear chromosome organization (Fig. 2A). Furthermore, the KEGG analysis demonstrated that these genes play a pivotal role in key cellular processes, including the regulation of the cell cycle, DNA replication, and the signaling pathway involving Forkhead box O (FoxO) (Fig. 2B). These results offer important insights into the molecular pathways driving renal fibrosis in DKD and could help in identifying novel therapeutic targets for treatment.

### 4.4 Mendelian Randomization Analysis

**Fig. 3:**
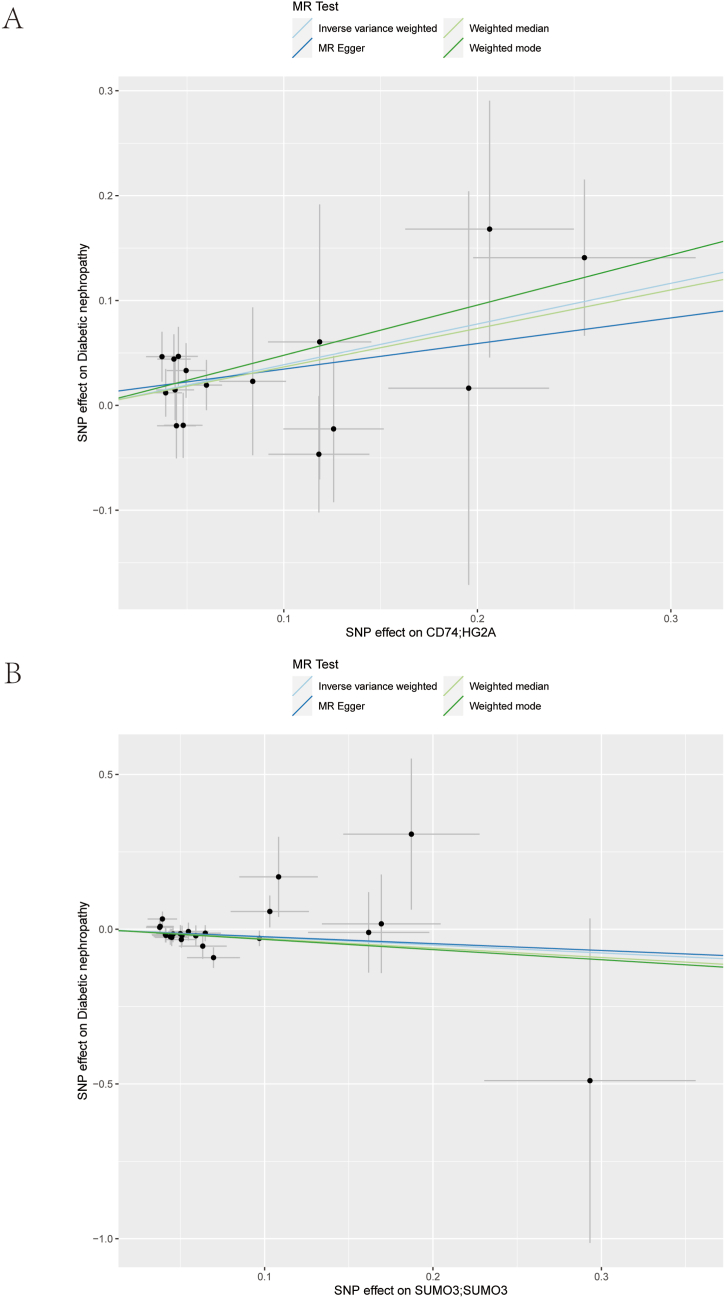
Mendelian Randomization Analysis. (A–B) Scatter plots depicting MR analysis of key genes. Different colors denote distinct statistical methods, while the slope of the line illustrates the causal effect of each method. MR: Mendelian randomization. (For interpretation of the references to color in this figure legend, the reader is referred to the Web version of this article.)

We utilized the 219 intersecting genes identified previously and accessed relevant data on diabetic kidney disease from the deCODE database. Utilizing aggregated statistical data from 312,650 samples (Controls: 308,539; Cases: 4111), we obtained the outcome ID finngen_R9_DM_NEPHROPATHY_EXMORE. Subsequently, we employed the extract_instruments and extract_outcome_data tools to establish 77 gene-outcome causal relationships (Supplementary Table 2). Mendelian randomization (MR) analysis identified two gene-outcome pairs with a positive causal association (Fig. 3A–B), with an inverse-variance weighted (IVW) p-value <0.05. These genes were *CD74* and *SUMO3*. The presence of one variant with an odds ratio (OR) of *SUMO3* (OR: 0.775; 95 % CI: 0.619–0.970; p = 0.044) potentially correlated with a reduced risk of diabetic kidney disease (Supplementary Table 3), while the *CD74* gene (OR: 1.474; 95 % CI: 1.128–1.926; p = 0.004) was linked to an elevated risk (Supplementary Table 4).

**Fig. 4:**
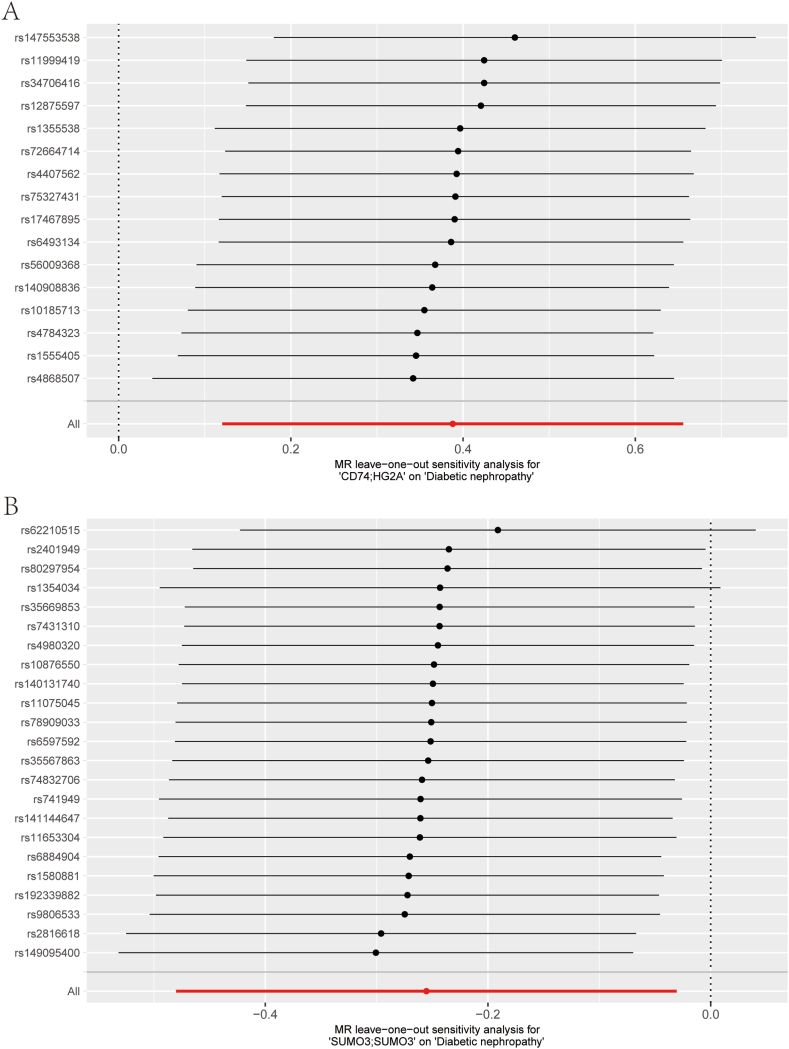
Leave-one-out Analysis (A–B) Forest plot illustrating the leave-one-out test results for SNPs corresponding to key genes. SNPs: single nucleotide polymorphisms.

To ensure the reliability of the causal relationships identified for these two genes, a leave-one-out sensitivity analysis was performed. The results revealed that the removal of any individual SNP did not significantly alter the overall confidence intervals, reinforcing the robustness of the established causal links (Fig. 4A–B). Additionally, we found that both *CD74* and *SUMO3* passed the heterogeneity test. Consequently, these two genes have been identified as prime candidates for further investigation.

### 4.5 Evaluation of immune infiltration

**Fig. 5:**
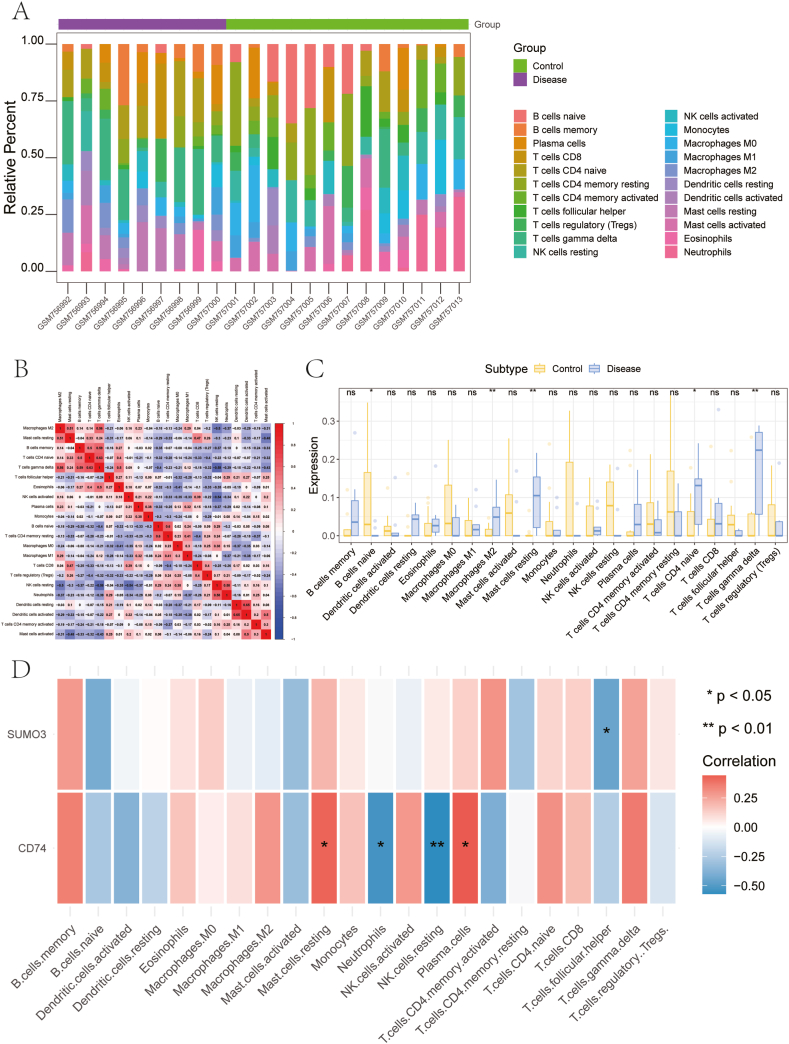
Immune Infiltration Analysis (A) Relative percentages of 22 immune cell subsets. (B) Pearson correlation between 22 types of immune cells, where blue indicates negative correlation and red indicates positive correlation. (C) Differences in immune cell content between DKD samples and calcitriol-treated samples. (D) Correlation of two key genes with immunity and immune cells. (For interpretation of the references to color in this figure legend, the reader is referred to the Web version of this article.)

The analysis focused on immune cell infiltration patterns in both the DKD cohort and the control group, aiming to clarify the interplay between key genes and the molecular pathways they might affect throughout the progression of the disease. We quantified immune cell proportions for each patient and analyzed their interactions (Fig. 5A–B). The analysis identified a marked rise in M2 macrophages, resting mast cells, naive CD4^+^ T cells, and gamma delta T cells in patients with DKD relative to non-diseased controls (Fig. 5C).

**Fig. 6:**
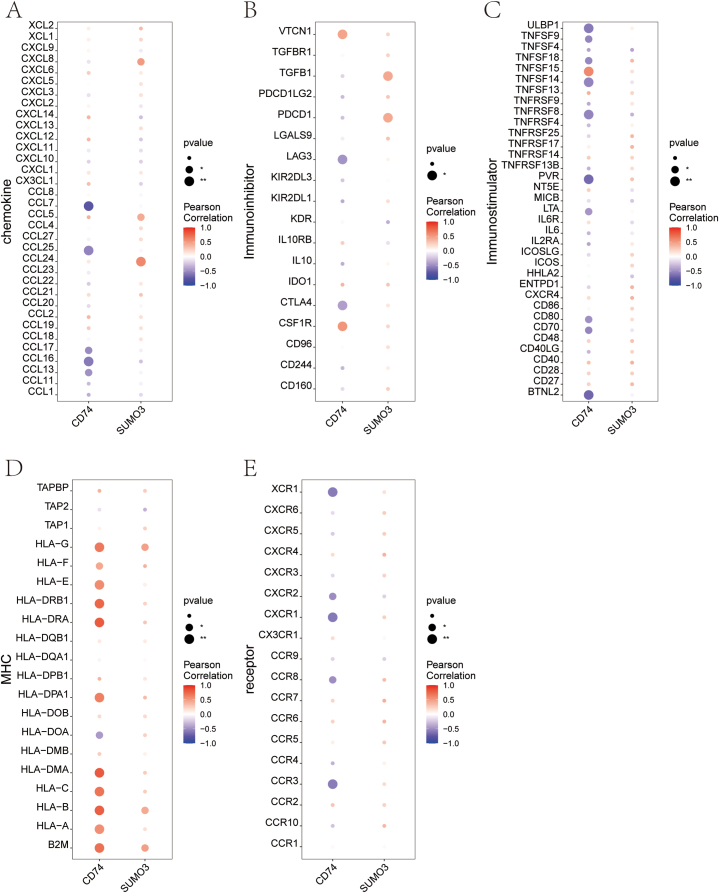
Relationship Between Key Genes and Immune Factors. (A–E) Correlation of key genes with chemokines, immunoinhibitors, immunostimulators, MHC, and receptors. MHC: Major Histocompatibility Complex.

Furthermore, the connection between significant genes and immune cell infiltration in a DKD dataset was explored to uncover potential molecular pathways that may be influenced by these genes in disease progression. *CD74* displayed a strong positive association with resting mast cells and plasma cells, while demonstrating a negative correlation with neutrophils and resting NK cells. On the other hand, *SUMO3* was significantly inversely correlated with follicular helper T cells (Fig. 5D). To deepen our understanding of these interactions, data from the TISIDB database were utilized to examine the connections between key genes and various immune-related elements, including immunosuppressive agents, immune stimulators, chemokines, and their corresponding receptors. These results emphasize the pivotal role of key genes in modulating immune cell infiltration, revealing their broader influence on the immune dynamics within the disease microenvironment (Fig. 6A–E).

### 4.6 Enrichment analysis via GSEA and GSVA

**Fig. 7:**
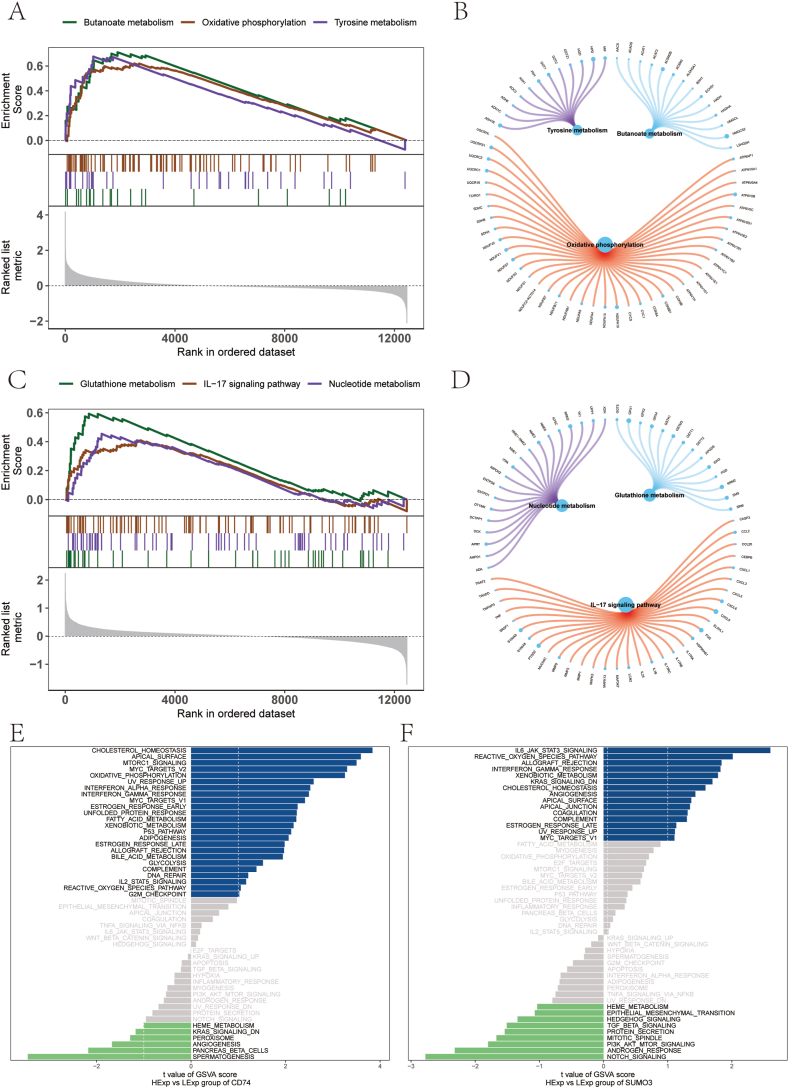
GSEA and GSVA Pathway Enrichment Analysis. (A–B) GSEA demonstrates significant pathway enrichment for CD74. (C–D) GSEA indicates pathway enrichment for SUMO3. (E) GSVA analysis based on CD74 expression levels. (F) GSVA analysis based on SUMO3 expression levels. GSEA: Gene Set Enrichment Analysis; GSVA: Gene Set Variation Analysis.

To gain a deeper understanding of the molecular mechanisms underlying DKD progression, we conducted an in-depth analysis of the signaling pathways enriched by the two key genes. Through Gene Set Enrichment Analysis (GSEA), we identified a significant association between *CD74* and multiple critical pathways, including butanoate metabolism, oxidative phosphorylation, and tyrosine metabolism (Fig. 7A–B). Conversely, *SUMO3* showed enrichment in pathways such as glutathione metabolism, IL-17 signaling, and nucleotide metabolism (Fig. 7C–D). Additionally, Gene Set Variation Analysis (GSVA) analysis unveiled that elevated *CD74* expression is linked to pathways such as cholesterol homeostasis, mTORC1 signaling, and oxidative phosphorylation (Fig. 7E), while increased *SUMO3* expression correlates with pathways such as IL-6/JAK/STAT3 signaling, xenobiotic metabolism, and KRAS signaling down-regulation (KRAS_SIGNALING_DN) (Fig. 7F). These findings indicate that the critical genes could impact disease development by engaging with these essential molecular processes.

### 4.7 Analysis of transcriptional regulation and gene correlation

**Fig. 8:**
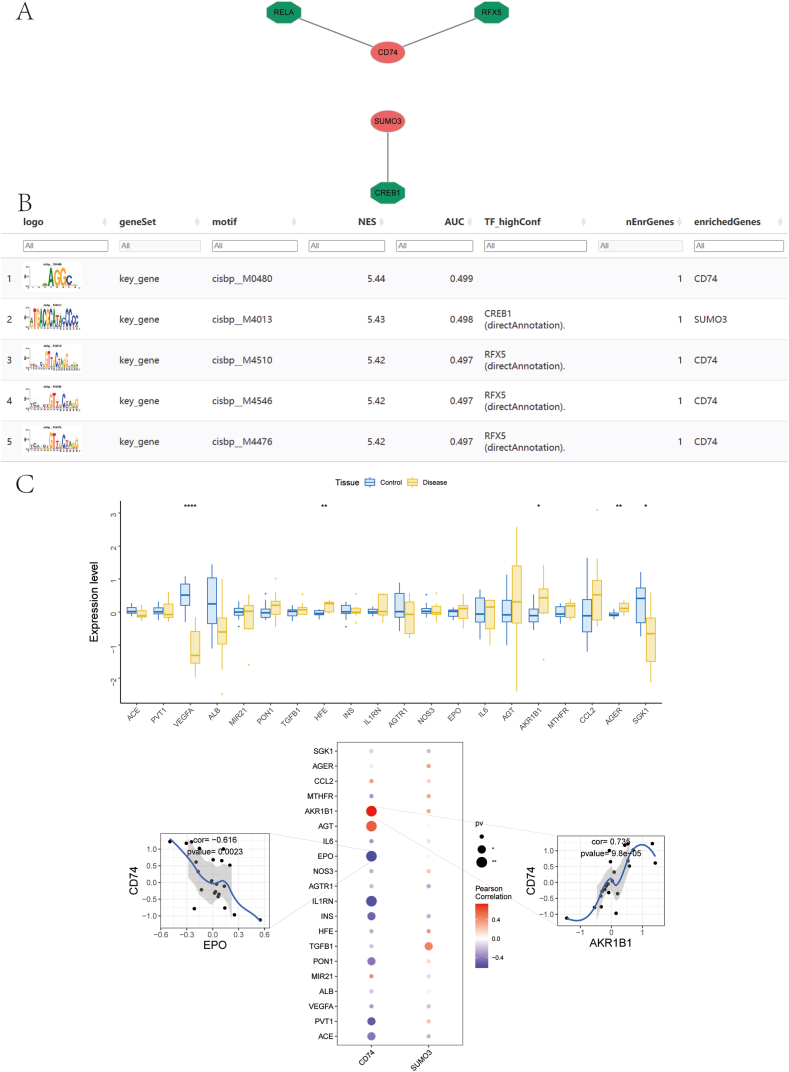
Transcriptional Regulation and Correlation Analysis of Key Genes. (A) Transcriptional regulation network of key genes, with key genes shown in red and transcription factors in green. (B) Display of all enriched motifs and their corresponding transcription factors related to the key genes. (C) Upper panel: expression differences of disease-associated genes, with blue representing diabetic kidney disease (DKD) and yellow representing controls. Lower panel: correlation analysis between disease-associated genes and key genes, where blue indicates a negative correlation and red indicates a positive correlation. (For interpretation of the references to color in this figure legend, the reader is referred to the Web version of this article.)

The results presented in Fig. 8A illustrate a gene regulatory network centered around *CD74*, with its connections to key transcription factors such as *RELA*, *RFX5*, *SUMO3*, and *CREB1*. Notably, *CD74* appears to be central, potentially playing a crucial role in gene regulation pathways involving these transcription factors. *SUMO3* and *CREB1* are also involved, further suggesting a layered regulatory mechanism influencing CD74's function. In Fig. 8B, motif enrichment analysis is shown, providing evidence of enriched transcription factor binding motifs in key genes. The top motifs include cisbp_M0480, cisbp_M4013, and cisbp_M4510, all with normalized enrichment scores (NES) above 5.4. The associated AUC values, although modest, further support the relevance of these motifs in transcriptional regulation. The transcription factors *CREB1* and RFX5 are identified with high confidence through direct annotation, implying strong evidence for their involvement in the regulation of *CD74* and *SUMO3*. These findings underscore the importance of *CD74* as a central regulatory node in this gene set, potentially influencing immune response or other critical pathways in disease contexts such as DKD.

Additionally, a correlation matrix in the middle panel reveals the relationships between *CD74*, *SUMO3*, and several DKD-related genes. *CD74* shows a strong positive correlation with AKR1B1 (R = 0.735, p = 9.8e-05), while exhibiting a significant negative correlation with EPO (R = −0.616, p = 0.0023) (Fig. 8B). These findings align with previous data from the GeneCards database, which identified *AKR1B1* and other genes, such as *VEGFA*, *HFE*, *AGER*, and *SGK1*, as having distinct expression differences across patient groups. The correlations between *CD74*, *AKR1B1*, and *EPO* suggest that *CD74* may modulate both oxidative stress pathways (AKR1B1) and erythropoiesis (EPO), indicating its dual role in pro-inflammatory and protective mechanisms within DKD. The findings emphasize *CD74* as a central player in the immune and inflammatory response, making it a promising target for future research on DKD pathogenesis and therapeutic intervention strategies.

### 4.8 Diagnostic Value and Clinical Correlation of Key Genes

**Fig. 9:**
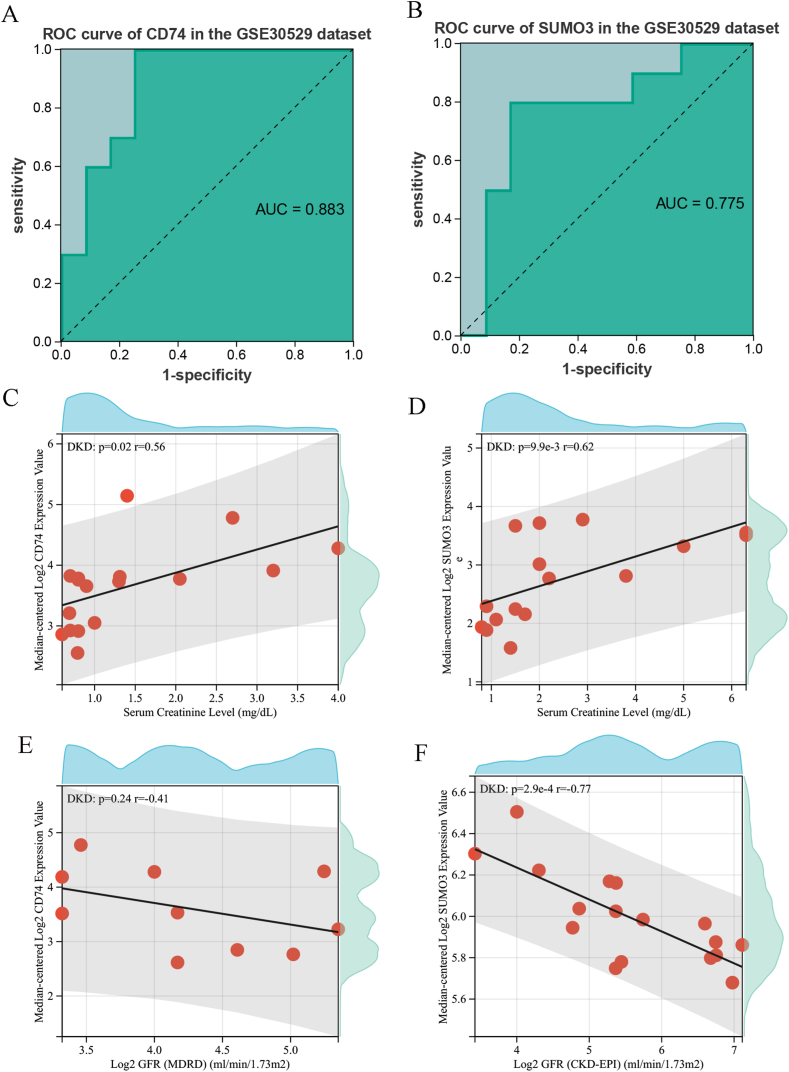
Diagnostic Value and Clinical Correlation of Key Genes. (A–B) ROC curves for key genes in the independent dataset GSE30529. (C–D) Scatter plots showing the correlation between key genes and GFR. (E–F) Scatter plots showing the correlation between key genes and Scr levels. ROC: Receiver Operating Characteristic; GFR: glomerular filtration rate; Scr: serum creatinine.

This study investigates the diagnostic potential and clinical relevance of key genes in DKD, utilizing the independent validation dataset GSE30529. To evaluate the ability of these genes to differentiate DKD from normal samples, a Receiver Operating Characteristic (ROC) curve analysis was conducted. Among the key findings, *SUMO3* (Area Under the Curve (AUC): 0.775) and *CD74* (AUC: 0.883) demonstrated strong diagnostic performance, with all key genes achieving an AUC greater than 0.70 (Fig. 9A–B). A subsequent Spearman rank correlation analysis highlighted a significant relationship between the expression of key genes and both adjusted glomerular filtration rate (GFR) and serum creatinine (Scr) levels. *CD74* and *SUMO3* were inversely correlated with GFR (r = −0.41, *p* = 0.24; r = −0.77, *p* = 2.9e-4, respectively) (Fig. 9C–D), while both showed a positive correlation with Scr levels (r = 0.56, *p* = 0.02; r = 0.62, *p* = 9.9e-3, respectively) (Fig. 9E–F). These results underscore the potential of these key genes as diagnostic biomarkers for DKD, providing valuable insights into their role in renal impairment and disease progression, as validated through the GSE30529 dataset.

### 4.9 Expression of key genes in single cells and HPA database verification

**Fig. 10:**
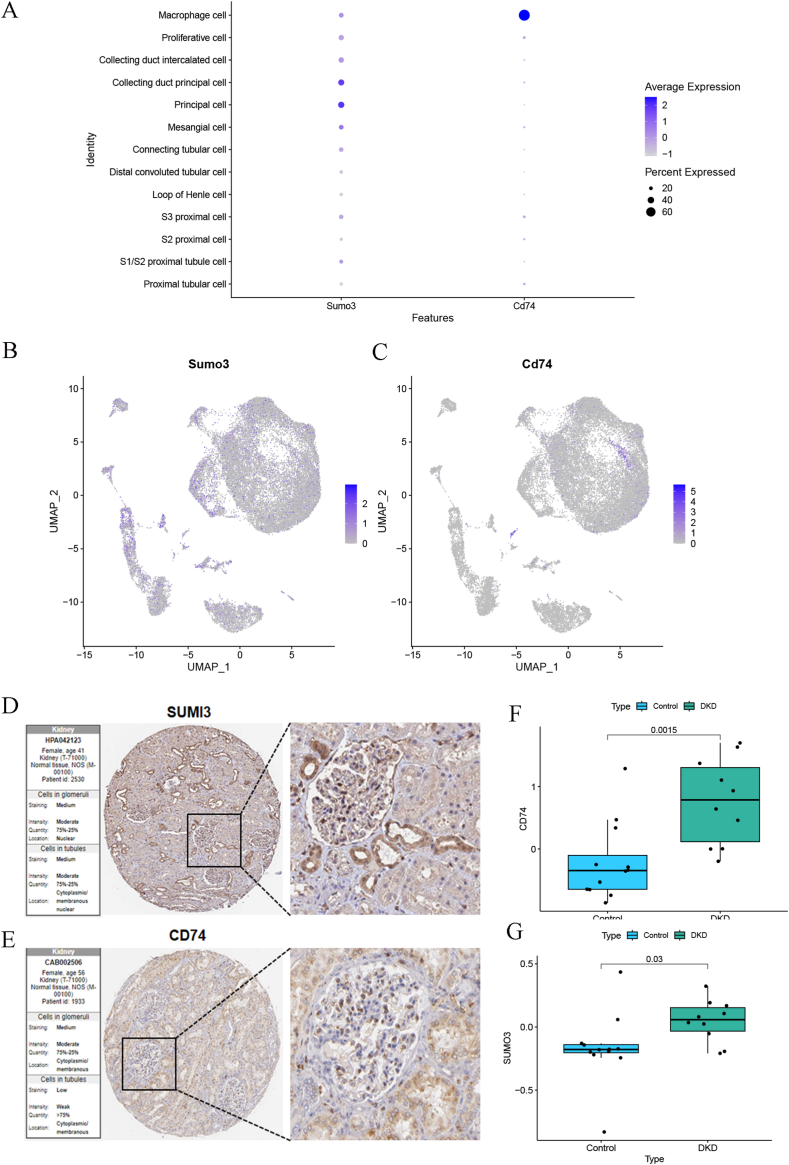
Expression of key genes in single cells and HPA database verification. (A–C) Expression profiles of key genes in 13 cell types. (D–E) Immunohistochemical staining of SUMO3 and CD74 proteins in normal renal tissues from the Human Protein Atlas database. (F–G) Analysis of key gene expression in the independent validation dataset GSE30529, showing significant differences between DKD and control groups.

When evaluating biomarkers for disease diagnosis, it is crucial to consider both their differential expression between healthy individuals and those with the disease, as well as their diagnostic effectiveness. In this context, we conducted an analysis to determine the suitability of the key genes for diagnostic purposes. The expression patterns of the key genes were mapped across 13 distinct cell types (Fig. 10A–C), providing a comprehensive view of their distribution within the cellular landscape. In addition, we conducted a co-expression analysis, focusing on the interaction between these key genes and others known to impact disease progression at the single-cell level. The top five co-expressed genes are highlighted in Supplementary Figs. 2 and 3, offering insights into potential gene networks that may contribute to the pathophysiology of the condition. Using the AUCell function, we quantitatively analyzed the activity differences between immune and metabolic pathways in single-cell data, revealing variations in activity levels between key genes and these pathways (Supplementary Fig. 4).

The Human Protein Atlas (HPA) database provided immunohistochemical profiles of the two core genes, SUMO3 and CD74, at the protein level. In normal renal tissues, SUMO3 exhibited strong staining in glomeruli and renal tubules, while CD74 translation protein displayed strong staining in glomeruli and moderate staining in renal tubules (Fig. 10D–E).

Furthermore, an examination of the independent validation dataset GSE30529 uncovered significant differences in the expression of key genes between DKD group and healthy controls (p < 0.05), with elevated expression detected in the DKD group (Fig. 10F–G).

## 5 Discussion

Despite advances in research, there remains a significant gap in effective therapeutic strategies to manage and slow the progression of DKD. The challenge lies in the complexity of its pathophysiology, which requires a multifaceted approach to develop more targeted and successful interventions. Although evidence has suggested that active vitamin D has a positive impact on the treatment of DKD, the specific molecular mechanisms underlying its effects remain to be further elucidated. This research combines scRNA-seq with traditional bulk RNA sequencing, complemented by Mendelian randomization analysis, to identify key genes responsible for the therapeutic effects of active vitamin D in diabetic kidney disease (DKD). Through this integrative approach, the study seeks to unravel the molecular pathways that mediate these effects, providing a more comprehensive understanding of how active vitamin D modulates DKD progression. By examining gene expression at both single-cell and bulk tissue levels, the research aims to offer novel insights into potential molecular targets for improved therapeutic interventions.

Kidney fibrosis is a pivotal driver in the progression of DKD toward end-stage renal failure [17]. This condition is characterized by the excessive buildup of extracellular matrix (ECM) in renal tissues, which disrupts the normal architecture and impairs kidney function. Over time, these structural changes culminate in the formation of scar tissue, further compromising renal function and accelerating the decline toward renal failure. This process is marked by inflammation, cell death, and injury to the renal tubular interstitium [18]. Recognizing biomarkers for the early detection, treatment, and prognosis of DKD is essential [19,20]. Noninvasive evaluation methods, such as magnetic resonance imaging (MRI), ultrasound, and terahertz biomedical detection, have demonstrated potential in assessing renal fibrosis [21]. Additionally, long non-coding RNAs have gained recognition as potential therapeutic targets in renal fibrosis, providing new avenues for treatment [22]. In this research, utilizing single-cell transcriptomic data from DKD rats treated with active vitamin D, we identified 11 unique cellular subpopulations. Notably, in the active vitamin D group, the proliferative cell subpopulation showed a marked reduction compared to the control group. The association between proliferative cells and fibrosis in DKD has been extensively studied. Senescent renal tubular epithelial cells have been discovered to activate fibroblasts through the secretion of Shh, thereby fostering the progression of DKD and contributing to renal fibrosis [23]. Additionally, the presence of proliferative lupus nephritis (LN) has been associated with the worst renal fibrosis and tubular atrophy, further emphasizing the link between proliferative cells and fibrosis [24]. Furthermore, in DKD, both myofibroblast transdifferentiation and cell proliferation have been recognized as significant factors contributing to renal fibrosis [25]. These findings underscore the intricate relationship between proliferative cells and the advancement of fibrosis in diabetic kidney disease. By screening genes through databases related to renal fibrosis, two core genes, SUMO3 and CD74, were identified to have a causal relationship with DKD, highlighting their strong predictive accuracy in the validation dataset. In summary, the discovery of biomarkers and the development of noninvasive evaluation methods are crucial for the early diagnosis and management of renal fibrosis in DKD. These advancements offer potential avenues for targeted therapeutic interventions and improved prognostic assessment in DKD.

Two core biomarkers, *SUMO3* and *CD74*, have been found to be closely linked with fibrosis in DKD. *SUMO3*, a small ubiquitin-like modifier, plays a key role in regulating numerous cellular functions, such as modulating the cell cycle, assisting in DNA repair processes, and fine-tuning gene expression [26]. Two core biomarkers, *SUMO3* and *CD74*, have been found to be closely linked with fibrosis in DKD. *SUMO3*, a small ubiquitin-like modifier, plays a key role in regulating numerous cellular functions, such as modulating the cell cycle, assisting in DNA repair processes, and fine-tuning gene expression [29]. This modification can alter protein stability, localization within the cell, and interaction networks, ultimately impacting critical cellular pathways including transcription, DNA repair, and signal transduction [27]. This modification can alter protein stability, localization within the cell, and interaction networks, ultimately impacting critical cellular pathways including transcription, DNA repair, and signal transduction [[28], [29], [30], [31]]. This modification can alter protein stability, localization within the cell, and interaction networks, ultimately impacting critical cellular pathways including transcription, DNA repair, and signal transduction [32]. However, the connection between *SUMO3* and fibrosis in DKD is still underexplored, highlighting the need for further study.

*CD74*, a cell surface receptor, interacts with macrophage migration inhibitory factor (MIF), playing a crucial role in regulating immune responses and inflammatory pathways. Upon binding to MIF, *CD74* triggers signaling cascades that control immune cell migration, antigen processing, and cytokine secretion. This protein is essential not only for maintaining immune homeostasis but also for the development and persistence of inflammatory responses, making it a key player in both normal immune function and the pathology of various inflammatory and autoimmune diseases [33]. *CD74* has been implicated in a range of inflammatory conditions, including liver fibrosis [34], kidney disorders [35], and Alzheimer's disease [36]. Furthermore, it is associated with autosomal dominant polycystic kidney disease (ADPKD), where it contributes to disease progression, particularly through its involvement in renal interstitial inflammation and fibrosis [37].These findings underscore the critical roles of *SUMO3* and *CD74* in the pathogenesis of DKD fibrosis, offering valuable insights into their potential as therapeutic targets in fibrosis-related kidney diseases.

In DKD, the involvement of immune cells is crucial in driving renal inflammation and fibrosis. A growing body of research has emphasized the role of various immune cell types, such as macrophages, mast cells, and T cells, in contributing to the progression of DKD([[38], [39], [40]]). Our study supports these findings by identifying significantly elevated levels of M2 macrophages, resting mast cells, naïve CD4 T cells, and γδ T cells in individuals with DKD compared to healthy controls. These observations highlight the critical role of these immune cell populations in the progression of DKD, suggesting their potential as therapeutic targets to mitigate disease progression. An important aspect of this study is the examination of the relationship between the key genes *CD74* and *SUMO3* and their interactions with immune cells, providing insights into their contributions to the pathophysiology of DKD. The observed positive correlation between *CD74* and both mast cells and plasma cells, along with its negative association with neutrophils and NK cells, suggests that *CD74* may influence immune cell activation and migration. Additionally, the inverse relationship between *SUMO3* expression and follicular helper T cells suggests that SUMOylation may play a role in modulating T cell-driven immune responses. GSEA and GSVA results have revealed signaling pathways that *CD74* and *SUMO3* may be involved in DKD. These findings align with the known metabolic disturbances and increased oxidative stress in DKD, offering new perspectives for understanding the pathophysiological mechanisms of the disease. These findings offer new targets for future therapeutic strategies, particularly by modulating the function of immune cells and diminishing kidney inflammation and fibrotic progression. Research has identified immune-related biomarkers and regulatory elements as well as possible treatment targets for DKD, emphasizing the immune system's significant influence on both the initiation and advancement of the disease [41,42].

This study comprehensively employed single-cell transcriptomics, genomics, immune infiltration analysis, and pathway enrichment analysis to delve into the molecular mechanisms and immunological microenvironmental characteristics of DKD. We successfully identified *CD74* and *SUMO3* as key genes in the progression of DKD and elucidated their potential roles in immune cell infiltration and metabolic pathways. Despite leveraging a range of bioinformatics tools and databases, this study has several limitations. Primarily, the analyses were conducted using in vitro data, and in vivo experimental validation was not performed, which limits the scope of the findings. Secondly, our research was dependent on samples from public databases, which may present issues with sample selection bias and heterogeneity. Future research should include: 1) validating the functionality of key genes in animal models; 2) validating the association between key genes and DKD progression using clinical sample data for further confirmation; 3) exploring the upstream regulatory factors and downstream effector molecules of key genes to fully understand their mechanisms of action in DKD.

Conclusion: Based on the results above, we draw four conclusions: l) Our research performed an in-depth analysis of single-cell sequencing and large-scale RNA datasets to investigate how active vitamin D treatment influences cellular subpopulations and gene expression profiles in patients with DKD. 2)The results revealed that active vitamin D intervention significantly modulates the cellular subpopulation structure and gene expression profiles, particularly reducing proliferative cell subpopulations. 3)Enrichment analyses identified the FoxO signaling pathway as pivotal, while Mendelian randomization analysis pinpointed *SUMO3* and *CD74* as potential markers.

Moreover, *SUMO3* and *CD74* exhibited a strong connection to immune cell recruitment and the regulation of gene expression related to the disease, influencing multiple signaling pathways that drive its progression. 4) These results provide fresh insights into the possible mechanisms through which active vitamin D acts in DKD treatment and highlight *SUMO3* and *CD74* as potential biomarkers for therapeutic strategies in DKD.

## Data availability statement

The datasets utilized in this study were derived from publicly available sources, including GEO database entries GSE30528 (https://www.ncbi.nlm.nih.gov/geo/query/acc.cgi?acc=GSE30528) and GSE30529 (https://www.ncbi.nlm.nih.gov/geo/query/acc.cgi?acc=GSE30529), alongside fibrosis-related gene sets obtained from the GeneCards repository (https://www.genecards.org/). The sequencing data supporting the findings have been deposited in the NCBI Sequence Read Archive (SRA) under BioProject ID PRJNA1152282 (https://www.ncbi.nlm.nih.gov/bioproject/PRJNA1152282), with the raw sequence reads accessible via SRA accession numbers SRR30363421 and SRR30363420. Additional data and code from this study are available upon request from the corresponding author.

## Funding

This research received financial support from the Hubei Province Science and Technology Initiative (2022BCE065) and the Technology Transfer Program of 10.13039/501100016359Zhongnan Hospital, 10.13039/501100007046Wuhan University (2023CGZH-MS003).

## Ethics statement

The Ethics Committee of Minda Hospital, affiliated with Hubei Minzu University, granted approval for the study (Ethics Approval Number: Y2022009).

## CRediT authorship contribution statement

**MingXia Zhang:** Writing – review & editing, Writing – original draft, Visualization, Project administration, Data curation. **Mi Tao:** Writing – review & editing, Visualization, Validation, Supervision, Software, Resources, Project administration. **Quan Cao:** Visualization, Validation, Project administration. **Yousheng Cai:** Visualization, Formal analysis, Data curation. **Lin Ding:** Visualization, Data curation. **Zhenni Li:** Methodology, Data curation, Conceptualization. **Wen Chen:** Formal analysis, Data curation. **Ping Gao:** Writing – review & editing, Writing – original draft, Validation, Supervision, Resources, Formal analysis, Data curation, Conceptualization. **Lunzhi Liu:** Writing – review & editing, Writing – original draft, Validation, Project administration, Methodology, Formal analysis, Data curation, Conceptualization.

## Declaration of competing interest

The authors declare the following financial interests/personal relationships which may be considered as potential competing interests:Liu lunzhi reports a relationship with Minda Hospital of Hubei Minzu University that includes: non-financial support. MingXia Zhang reports a relationship with Minda Hospital of Hubei Minzu University that includes: non-financial support. Zhenni Li reports a relationship with Minda Hospital of Hubei Minzu University that includes: non-financial support. Ding Lin reports a relationship with Minda Hospital of Hubei Minzu University that includes: non-financial support. Ping Gao reports a relationship with 10.13039/501100016359Zhongnan Hospital of Wuhan University that includes: non-financial support. Mi Tao reports a relationship with 10.13039/501100016359Zhongnan Hospital of Wuhan University that includes: non-financial support. Quan Cao reports a relationship with 10.13039/501100016359Zhongnan Hospital of Wuhan University that includes: non-financial support. Yousheng Cai reports a relationship with 10.13039/501100007046Wuhan University School of Pharmaceutical Sciences that includes: non-financial support. Wen Chen reports a relationship with 10.13039/501100016359Zhongnan Hospital of Wuhan University that includes: non-financial support. Gaoping has patent pending to none. None If there are other authors, they declare that they have no known competing financial interests or personal relationships that could have appeared to influence the work reported in this paper.
