# Supplementary material for: Identification of crucial genes and possible molecular pathways associated with active vitamin D intervention in diabetic kidney disease
Source: Heliyon. 2024 Sep 25;10(19):e38334. doi: 10.1016/j.heliyon.2024.e38334 (PMC11470520; doi:10.1016/j.heliyon.2024.e38334)
Supplement: Multimedia component 1 [file mmc1.docx]

***Supplementary materials***


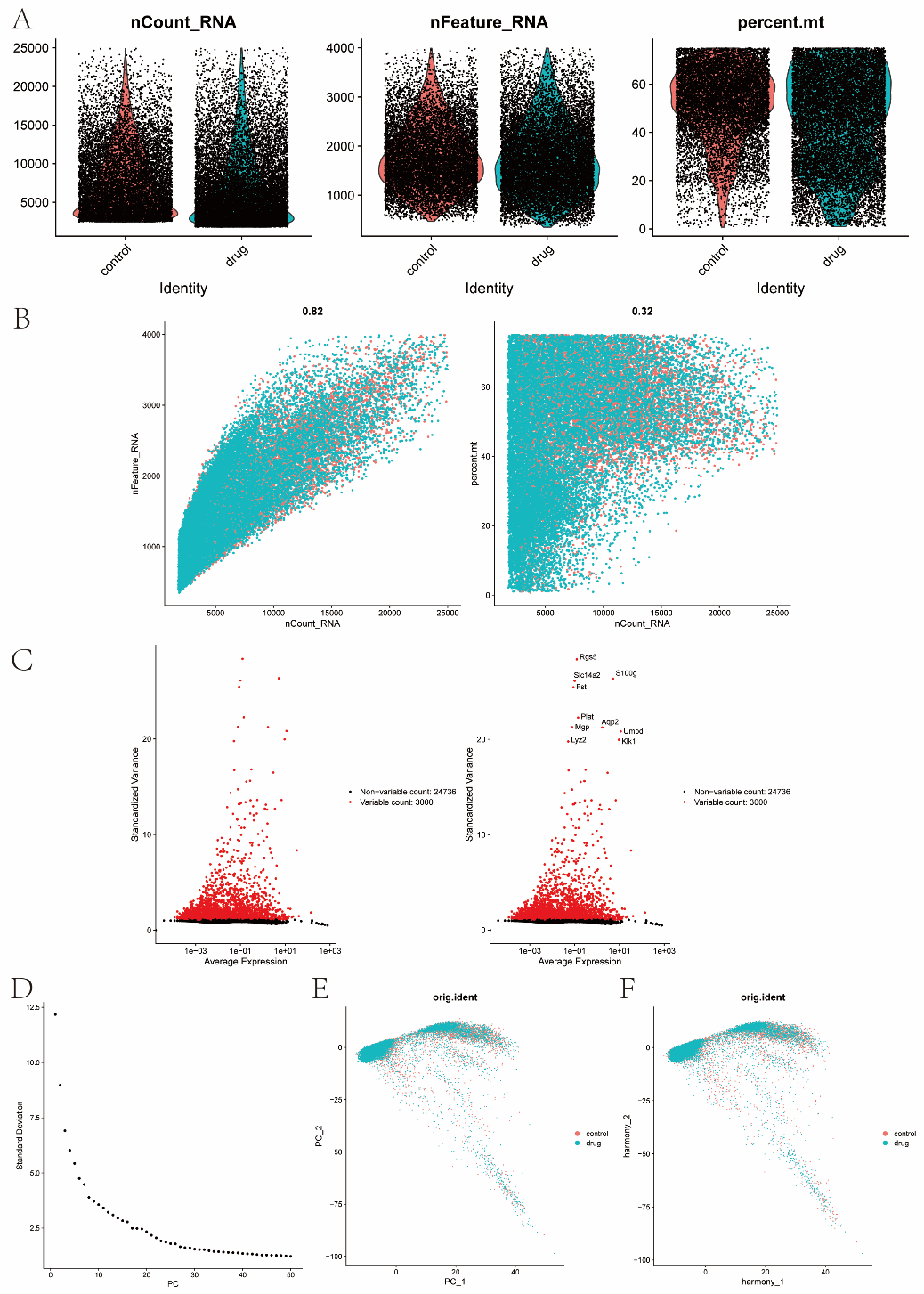


**Supplementary Figure 1 Single-cell pre-processing.**

(A) Quality control of single cells displaying cell count, gene count, and sequencing depth for each sample. (B) The left panel illustrates the correlation between cell sequencing depth and mitochondrial content, while the right panel depicts the relationship between sequencing depth and gene count, both showing a positive correlation.(C) Identification of significantly differentially expressed genes among cells, with characteristic variance plotted.(D) Ranking plot of variance for each principal component (PC).(E-F) Visualization of principal component analysis (PCA) and distribution of PCs, with cells represented by dots and samples distinguished by colors.


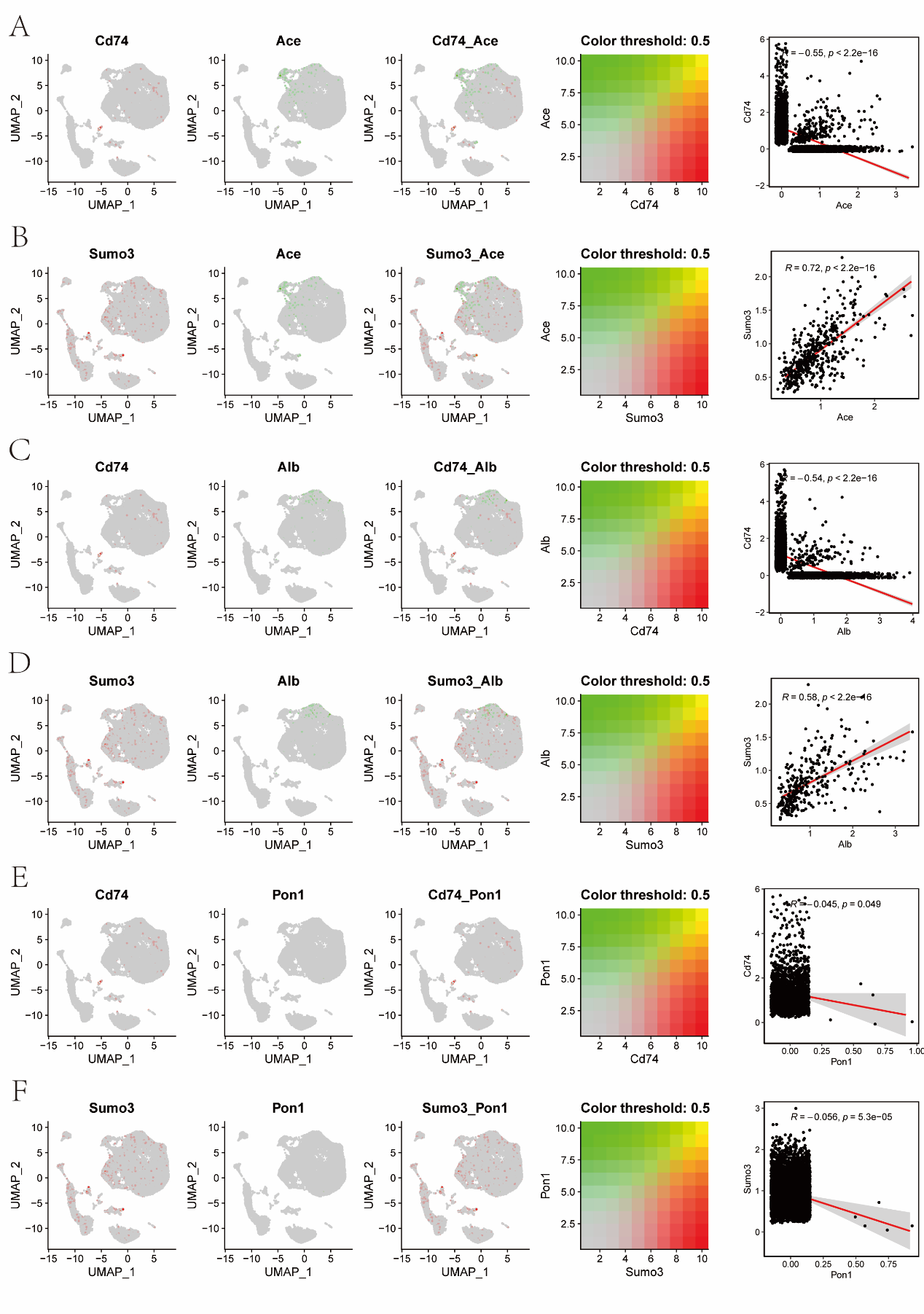


**Supplementary Figure 2: Co-expression analysis of key genes in single cells**

(A-F) Gene co-expression of disease genes and key genes in single-cell data, and co-expression gene correlations.


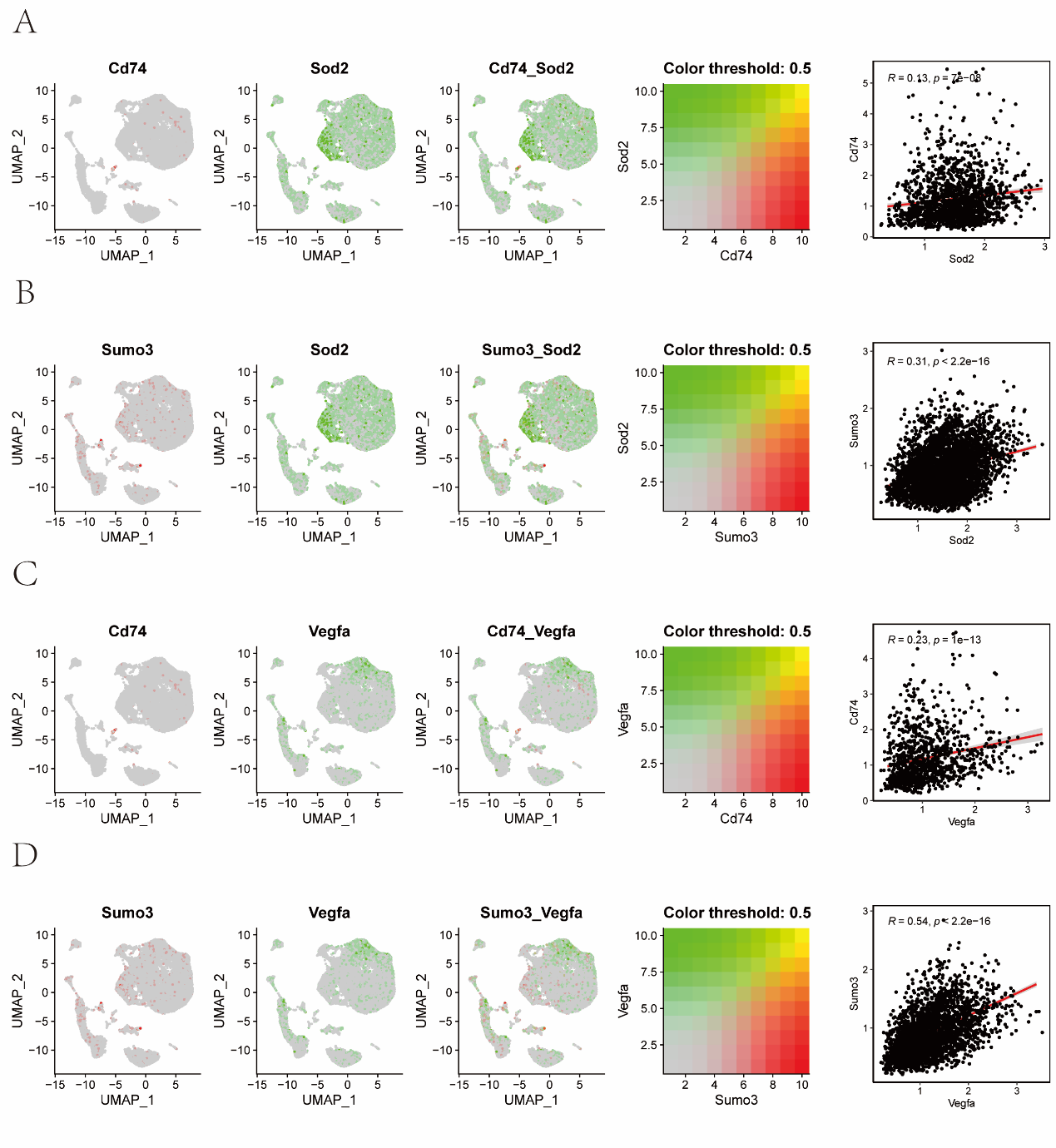


**Supplementary Figure 3: Co-expression analysis of key genes in single cells**

(A-D) Gene co-expression of disease genes and key genes in single-cell data, and co-expression gene correlations.


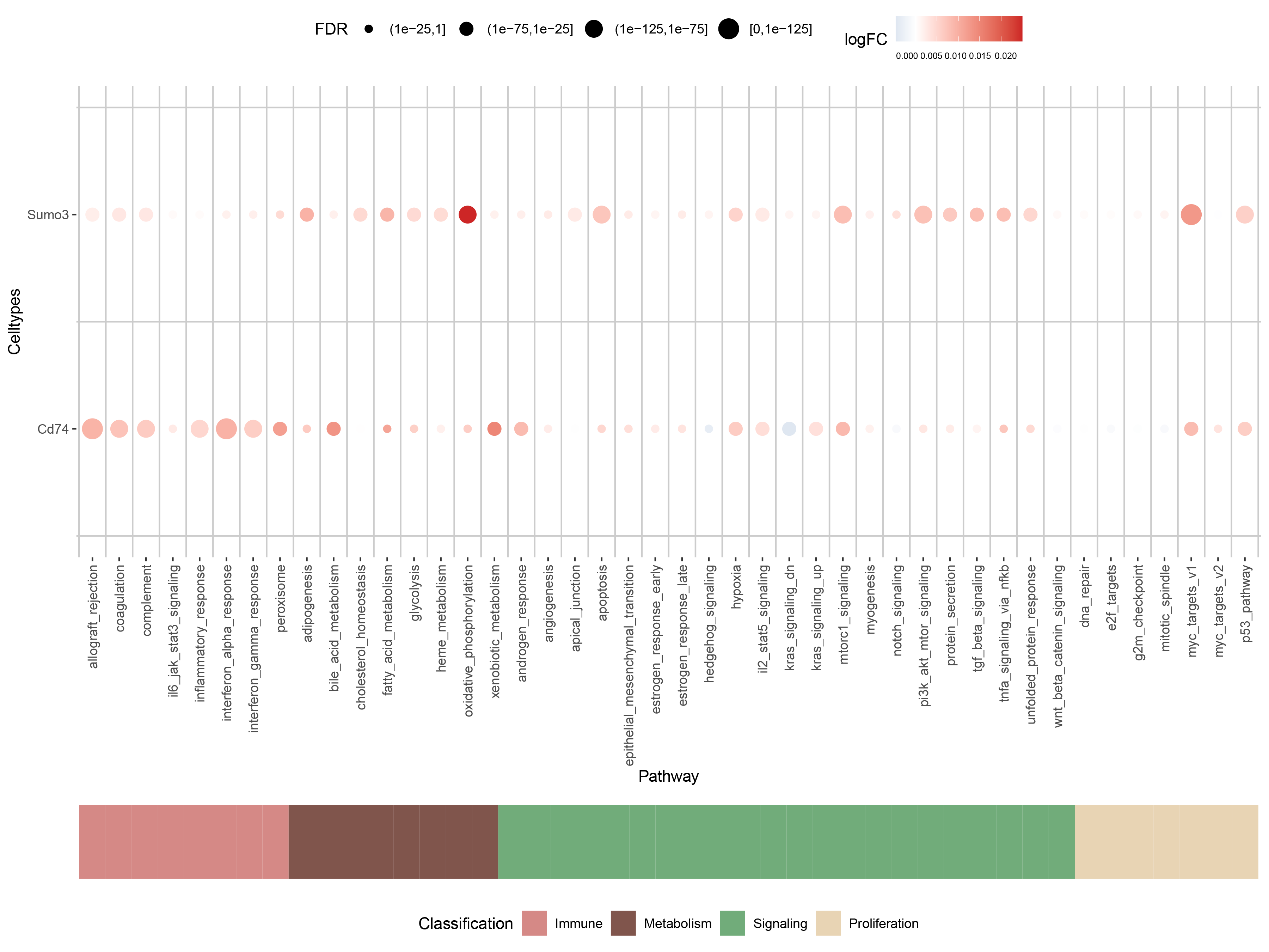


**Supplementary Figure 4**. Analysis of immune and metabolic pathway activities based on single-cell data.
